# Climate change and citrus fruit quality: links with plant physiology and mineral nutrition

**DOI:** 10.3389/fpls.2026.2009658

**Published:** 2026-10-22

**Authors:** Julia Morales, Belén Martínez-Alcántara, Ana Pérez-Piqueres, Isabel Rodríguez-Carretero, Ana Quiñones

**Affiliations:** Centre for Advanced Agricultural Technologies, Valencian Institute of Agricultural Research, Moncada, Valencia, Spain

**Keywords:** abiotic stress, biostimulants, carbon-nitrogen balance, priming, soil organic matter

## Abstract

Citrus production across growing regions is increasingly exposed to rising atmospheric CO_2_, higher temperatures, and greater variability in water availability, with Mediterranean orchards facing particularly pronounced climatic pressures. These climatic drivers affect citrus performance through interconnected changes in photosynthesis, root functioning, flowering, salinity responses, redox regulation, and source–sink relationships, with consequences for productivity and fruit quality. This review synthesises current knowledge on how climate change modifies citrus physiology and mineral nutrition, with particular attention to carbon–nitrogen coordination, carbohydrate partitioning, and nutrient allocation, and examines strategies to strengthen soil–plant functioning and nutritional resilience under increasing climatic variability. Temperature effects depend on exposure conditions: warmer soils may sustain root activity and prolong seasonal N uptake, whereas excessive temperatures can impair root functioning and nutrient acquisition, potentially altering coordination between vegetative and reproductive demand. Elevated CO_2_ may initially enhance carbon assimilation and water-use efficiency, but acclimation and changes in nitrogen allocation can limit this response. These alterations can affect fruit growth, ripening, composition, and susceptibility to physiological disorders, although their expression depends on stress intensity and timing, genotype, and developmental stage. The management discussion focuses on selected soil-management practices, biostimulants, and exogenous signalling compounds that may improve soil fertility and soil–root functioning or support physiological adaptation within established citrus orchards. This emphasis addresses opportunities to sustain existing plantings, including traditional cultivars, particularly where orchard renewal is economically constrained. Future progress will require multi-year field studies integrating soil, plant, climatic, and fruit-quality responses to assess the effectiveness and feasibility of these approaches under site-specific conditions.

## 1 Introduction

Global agriculture is currently facing unprecedented challenges driven by anthropogenic climate change, characterised by a continuous rise in atmospheric CO_2_ concentrations and a significant increase in global mean temperatures (IPCC, 2021; Elbasiouny et al., 2022). Current projections estimate that CO_2_ levels could reach between 800 and 1,000 ppm by the end of the 21st century, increasing surface temperatures by up to 5 °C (IPCC, 2021). In this context, the Mediterranean basin has emerged as a prominent climate-change hotspot, where temperatures are projected to rise approximately 20% faster than the global average, accompanied by an overall decline in precipitation of about 4% per degree of global warming (Lionello and Scarascia, 2018).

These changes pose challenges for Mediterranean citrus production, given the perennial and evergreen nature of citrus trees and their exposure to environmental stress across successive phenological stages (Jiménez-Jiménez et al., 2026). Combined heat, drought, and high-light stress can impair photosynthetic performance, although the activation of photosystem II repair mechanisms contributes to citrus tolerance (Balfagón et al., 2022). Damaging cold and freezing episodes remain relevant, with physiological responses depending on genotype, acclimation status, and exposure conditions (Vincent et al., 2020; Primo-Capella et al., 2021). Climate-related disruptions of soil–root functioning can also alter nutrient availability, uptake, and utilisation, thereby affecting tree nutritional status and productivity (Srivastava et al., 2026). Collectively, these physiological and nutritional disturbances may compromise citrus fruit development and quality. In particular, high temperatures can alter citrus fruit ripening and harvest timing (Mesejo et al., 2024), whereas water deficits during fruit development can restrict fruit growth and reduce final fruit size (García-Tejero et al., 2012).

Adaptation in established orchards therefore requires approaches that support both soil and plant functioning within existing scion–rootstock combinations. Integrated soil management practices, including mulching and organic matter management, can improve soil structure, water retention, and nutrient-use efficiency, thereby helping plants withstand drought conditions (Saputra et al., 2025). At the plant level, biostimulants may support stress mitigation or physiological preparedness, although protection following application does not necessarily demonstrate persistent priming (Cocetta et al., 2022). Understanding the effectiveness of these interventions may help sustain productive orchards where renewal is economically constrained or retaining traditional cultivars is a priority.

Previous reviews have addressed climate-related stresses, citrus nutrition, and adaptation strategies (Ziogas et al., 2021; Hussain et al., 2023; Gupta et al., 2024; Srivastava et al., 2026). The distinctive contribution of this review is its fruit-centred physiological perspective, linking climate-driven changes in carbon assimilation and root functioning with nutrient acquisition and allocation and their consequences for fruit growth, ripening, composition, and physiological disorders. Particular attention is given to the temporal coordination between carbon supply, nitrogen availability, and the changing demands of vegetative and reproductive organs, and to whether disruptions in this coordination could help explain fruit-quality responses. Although Mediterranean citrus production provides a particularly relevant context, evidence from other growing regions is included where it informs these relationships.

The management discussion focuses on opportunities to support the resilience of established citrus orchards through soil-management practices, biostimulants, and exogenous signalling compounds. These interventions are considered for their potential to improve soil fertility and soil–root functioning or support plant physiological responses within existing scion–rootstock combinations. This emphasis addresses adaptation where orchard renewal is economically constrained or retaining traditional cultivars is a priority. Rootstock and scion selection, irrigation-system design, and canopy management remain important adaptation tools, but their comprehensive evaluation falls outside the management scope of this review. The contribution of the selected interventions to sustainable adaptation is assessed alongside their efficacy, costs, input requirements, and practical feasibility. By connecting climate-driven physiological responses with nutritional dynamics and fruit outcomes, this review identifies mechanisms and knowledge gaps that are less apparent when these topics are considered separately.

## 2 Review design

This manuscript presents a narrative review of the effects of climate change on citrus fruit quality, with particular emphasis on the links between environmental conditions, plant physiology, and mineral nutrition. Relevant literature was identified from July to October 2026 through searches in Google Scholar and Web of Science, complemented by reference tracking of key publications and supplementary literature identification assisted by ChatGPT. The literature was selected to provide an integrated perspective on how climate-related changes in temperature, water availability and other environmental factors may affect citrus crops. Original citrus studies were prioritised for crop-specific conclusions, while evidence from other species and review articles was included to provide mechanistic or broader context and distinguished from direct citrus evidence. Studies were not restricted to Mediterranean production systems or mature field-grown trees; their interpretation considered experimental setting, plant age, genotype, stress intensity and duration, and phenological timing. For treatment studies, concurrent stress mitigation, protection following pretreatment, and evidence of persistence after treatment withdrawal were distinguished. For the management component, selection focused on soil-management practices, biostimulants, and exogenous signalling compounds relevant to adaptation within established plantings; a comprehensive assessment of orchard renewal, irrigation systems, or canopy management was outside this scope.

Searches were refined iteratively around combinations of terms relating to citrus, climate change, elevated CO_2_, heat, cold and freezing, drought, waterlogging, salinity, mineral nutrition, source–sink relationships, flowering, fruit quality, soil management, biostimulants, and stress priming. No restrictions on publication year were applied. Publications from the preceding five years were prioritised to capture recent advances, while older studies were retained where they provided relevant experimental evidence or foundational knowledge. AI-suggested references were checked against the original publications and assessed using the same relevance criteria as literature identified through other sources. **Figure 1** summarises literature identification and evidence appraisal. The review did not follow a prespecified systematic protocol and does not claim exhaustive coverage or a formal risk-of-bias assessment.

**Figure 1:**
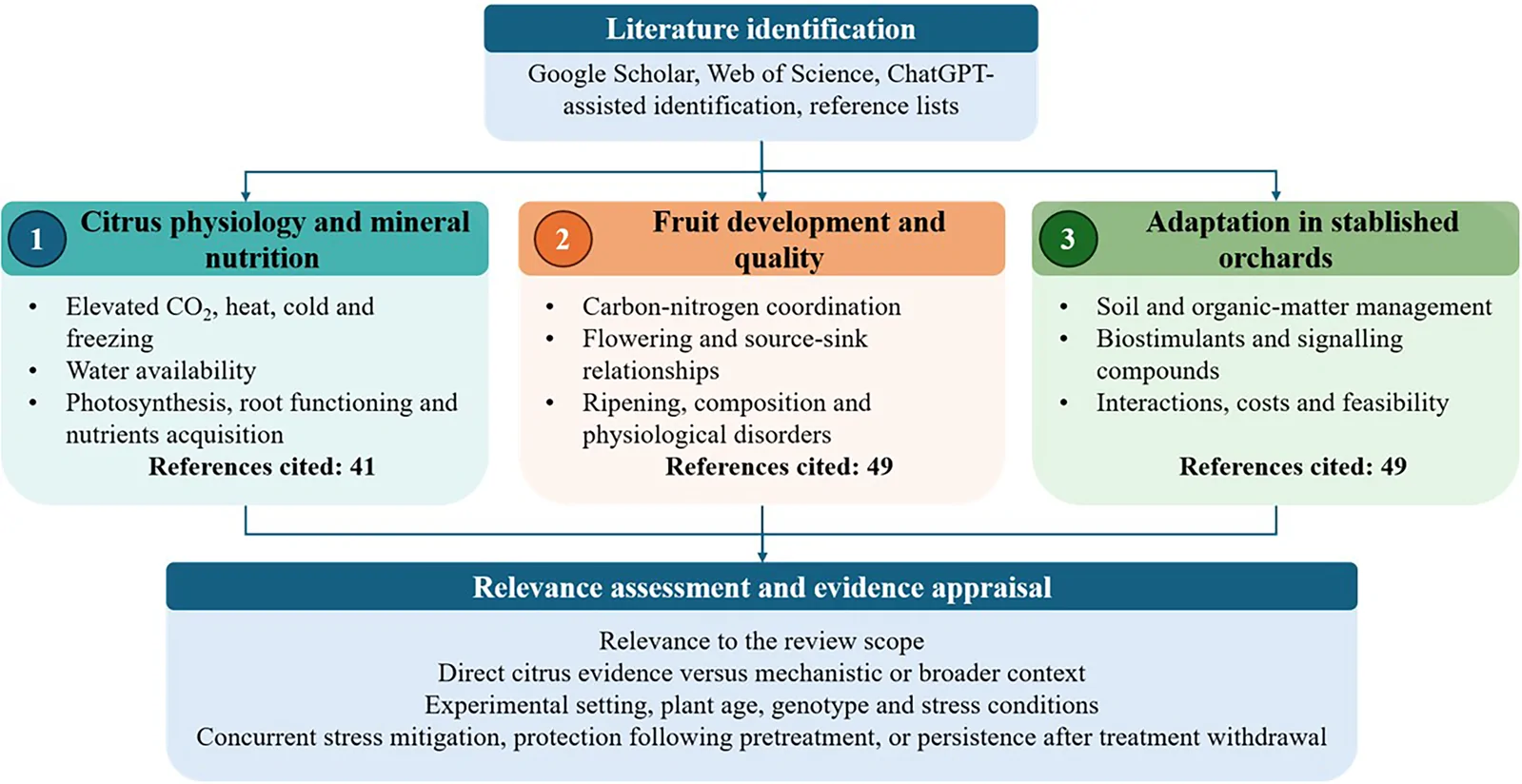
Thematic framework for literature identification and evidence appraisal in this narrative review. Counts indicate distinct references cited within each block; publications may contribute to more than one block.

## 3 Climate-driven changes in citrus physiology and nutrition

### 3.1 Atmospheric CO_2_ and high-temperature stress

Changes in atmospheric CO_2_ and temperature directly influence the physiological processes underlying carbon assimilation, water relations, and nutrient acquisition in citrus. Elevated CO_2_ can initially stimulate carbon assimilation in citrus by increasing CO_2_ availability for carboxylation and reducing photorespiration. This response may also increase photosynthetic water-use efficiency through reduced stomatal conductance and transpiration (Keutgen and Chen, 2001; Fischer et al., 2022). However, the magnitude and persistence of the CO_2_-induced stimulation of carbon assimilation depend on temperature, water and nutrient availability, genotype, and the capacity of sink organs to use or store the additional assimilated carbon (Mishra et al., 2019; Fischer et al., 2022). These potential short- and longer-term responses to elevated CO_2_, and their implications for carbon and nutrient allocation, are summarised in **Figure 2**.

**Figure 2:**
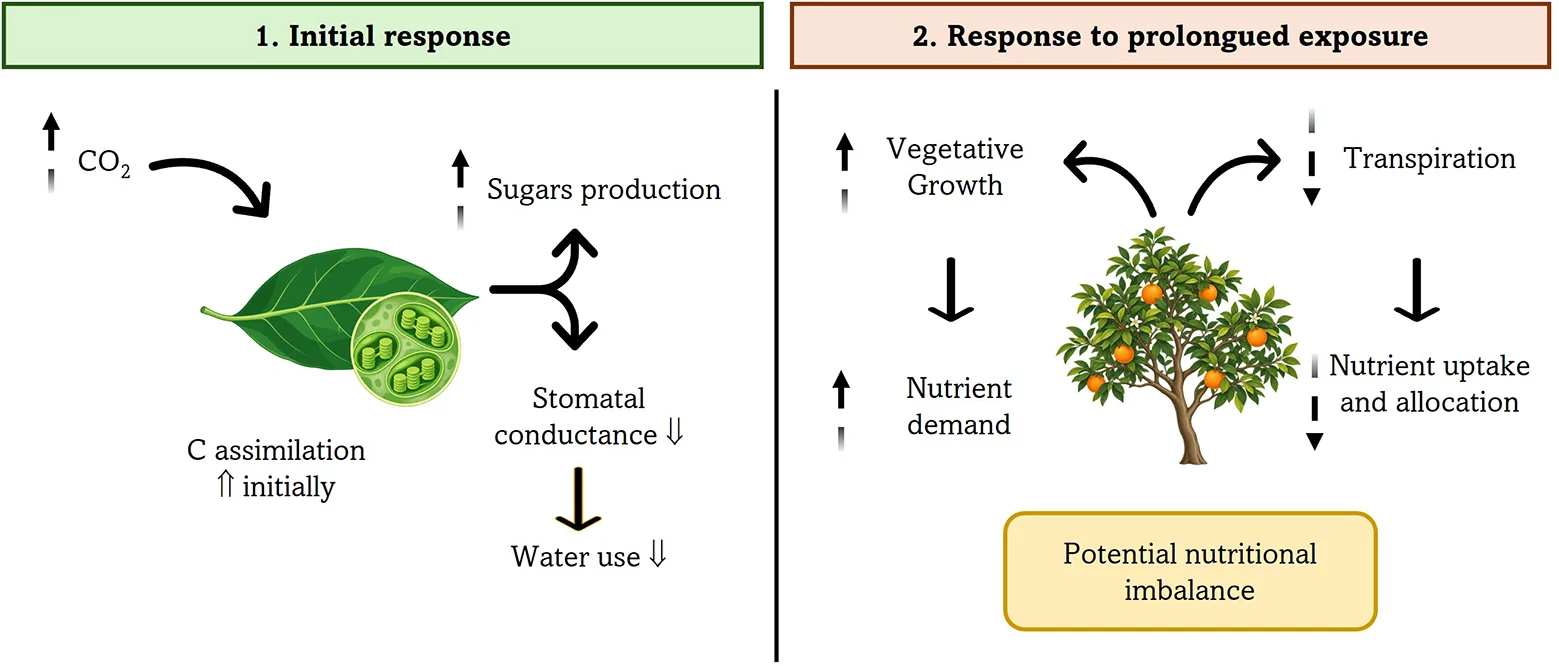
Conceptual overview of the initial and prolonged responses of citrus plants to elevated atmospheric CO_2_. This original conceptual illustration synthesises responses reported in citrus studies under elevated CO_2_ (Keutgen and Chen, 2001; Vu et al., 2002; Adam et al., 2004). The responses shown depend on genotype, exposure duration, temperature, and water and nutrient availability.

Prolonged exposure to elevated CO_2_ can further induce photosynthetic acclimation, involving a reduction in the amount of photosynthetic machinery required to sustain carbon assimilation. In young ‘Ambersweet’ orange trees, elevated CO_2_ increased photosynthetic rates despite reducing Rubisco activity and protein concentration, while total soluble protein remained unchanged. This response was interpreted as an optimisation of N use through the reallocation of N away from Rubisco towards other metabolic functions (Vu et al., 2002). Similarly, long-term CO_2_ enrichment resulted in photosynthetic down-regulation in mature sour orange trees grown under non-limiting N conditions, indicating that acclimation cannot necessarily be attributed to nitrogen deficiency (Adam et al., 2004). The magnitude of this response appears to vary among citrus species, leaf developmental stages, and experimental conditions. In *C. madurensis*, elevated CO_2_ increased plant growth, the shoot-to-root ratio, and leaf dry mass per unit area, but photosynthetic down-regulation occurred after 90 days at concentrations of 600 ppm and above (Keutgen and Chen, 2001). In drought-stressed lemon saplings, elevated CO_2_ similarly maintained higher photosynthetic rates and reduced stomatal conductance and water consumption, increasing wood carbon storage. However, its effects on Rubisco investment or whole-plant N allocation were not examined (Paudel et al., 2018). **Table 1** summarises the plant material, growing systems, exposure conditions, duration, and main variables measured in the selected studies.

**Table 1:** Experimental context and scope of inference of selected citrus studies on elevated CO_2_ and high-temperature stress.

Reference	Plant material and age	Growing system	Exposure and control	Duration	Main variables measured
Keutgen and Chen (2001)	Calamondin (*C. madurensis*) cuttings; 1-year greenhouse preculture	5-L containers with quartz sand; growth chambers	300 ppm reference versus 450, 600, 750 and 900 ppm CO_2_; 25/20 °C Day/night	90 days	Gas exchange, chlorophyll fluorescence, growth, leaf macronutrients and carbohydrates
Vu et al. (2002)	‘Ambersweet’ orange; 2 years old at initiation	Container-grown trees under controlled conditions	360 versus 720 ppm CO_2_; temperatures +1.5 and +6 °C above ambient	29 months	Gas exchange, water-use efficiency, Rubisco activity and protein content
Adam et al. (2004)	Sour orange (*C. aurantium*); adult trees after 14 years of experimental growth	In-ground trees in outdoor open-top chambers, Phoenix, USA	Ambient versus ambient +300 ppm CO_2_; no imposed warming; adequate water and nutrients	14 years; seasonal measurements in 2001–2002	Gas exchange, photosynthetic capacity, Rubisco, chlorophyll and woody biomass relationships
Paudel et al. (2018)	‘Villafranca’ lemon/*C. volkameriana*; 1 year old	7-L pots in CO_2_-controlled greenhouse compartments	400, 650 and 850 ppm CO_2_ × irrigated/drought treatments; 26/16 °C Day/night	4 months, including 1 month of drought and 1 month of rewatering	Gas exchange, water status and use, biomass, carbohydrates and leaf N, P and K
Balfagón et al. (2022)	Carrizo citrange and Cleopatra mandarin; 1-year-old seedlings	0.6-L pots in growth chambers	40 versus 23 °C; high light and water stress applied individually and in combinations	5 days of heat/high light; 24 h water stress; 24 h recovery	Gas exchange, leaf temperature, PSII function, leaf injury, transcriptomics and proteomics

These changes in carbon assimilation and biomass allocation also raise questions about mineral concentrations under elevated CO_2_. Across C_3_ plants, a meta-analysis reported reductions of 6.5–10% in the concentrations of P, K, Ca, S, Mg, Fe, Zn, and Cu, whereas Mn showed no significant change in the pooled analysis (Loladze, 2014). FACE data from major food crops further showed that responses differed among nutrients, species, and cultivars, indicating that carbohydrate dilution alone does not explain all compositional changes (Dietterich et al., 2015). In citrus, however, photosynthetic acclimation in *C. madurensis* was accompanied by a reduction in leaf N concentration, while the other macronutrients measured remained comparatively stable (Keutgen and Chen, 2001). Broader evidence of CO_2_-associated mineral dilution provides relevant context but does not establish a corresponding reduction in citrus fruit nutrient density.

In addition to its direct effects on citrus physiology, elevated atmospheric CO_2_ contributes to anthropogenic warming through increased radiative forcing (IPCC, 2021). The effects of rising CO_2_ and temperature on citrus should therefore be considered jointly. The interaction between rising temperature and elevated CO_2_ is complex, and their combined effects cannot be inferred simply by adding their individual effects. In young ‘Ambersweet’ orange trees, a growth regime 6 °C above ambient temperature reduced leaf water-use efficiency. However, elevated CO_2_ increased photosynthetic rates and compensated for this adverse temperature effect on water-use efficiency (Vu et al., 2002). Nevertheless, this compensatory response, observed under controlled conditions and in the absence of additional environmental constraints, cannot be directly extrapolated to the increasingly frequent combinations of heat, drought, and high irradiance occurring in Mediterranean orchards. Under these multifactorial stress conditions, high temperatures can contribute to a marked deterioration of citrus physiological functioning. In Carrizo citrange and Cleopatra mandarin, the combination of high temperature, drought, and high irradiance reduced photosynthetic activity, altered the redox balance of the electron transport chain, and compromised photosystem II integrity (Balfagón et al., 2022).

Temperature can also influence nutrient acquisition, although its effects depend on the exposure conditions. In one-year-old ‘Okitsu Wase’ satsuma trees grafted onto trifoliate orange, total acquisition of N, P, K, Ca, and Mg increased between constant growth temperatures of 15 and 25 °C but declined slightly at 30 °C over seven months (Inoue and Harada, 1988). Because citrus-specific evidence on mineral acquisition under severe heat and its interaction with elevated CO_2_ remains limited, studies in tomato provide additional mechanistic context. In tomato, six days at 42/37 °C day/night, with roots reaching 39 °C, reduced root-specific acquisition rates of N, P, K, and Fe (Giri et al., 2017). Elevated CO_2_ did not necessarily alleviate these nutritional constraints: in a separate tomato experiment using ^15^N labelling, the combination of 700 ppm CO_2_ and 38/33 °C reduced N acquisition, net translocation, and assimilation (Jayawardena et al., 2021). These studies provide complementary evidence on temperature-dependent mineral acquisition and its interaction with CO_2_, while the responses demonstrated in tomato require validation in citrus.

Beyond its effects on photosynthesis and nutrient acquisition, temperature also influences citrus reproductive development and the formation of fruit sinks. In citrus, floral induction responds to low temperatures or water-deficit conditions, depending on the growing environment, whereas floral differentiation occurs subsequently during bud sprouting (Agustí et al., 2022). In ‘satsuma’ mandarin, exposure under 15 °C promoted floral induction and increased *CiFT* expression, providing experimental evidence of temperature-responsive regulation of flowering (Nishikawa et al., 2007). Similar responses have been observed across a wide range of citrus species, including mandarins, sweet orange and even more distantly related taxa such as finger lime (Mesejo et al., 2022; Munoz-Fambuena et al., 2011; Marzal et al., 2026), suggesting that low-temperature mediated floral induction is a conserved trait within the Citrus genus and its relatives. Prolonged warm conditions during autumn and winter may therefore shorten exposure to inductive temperatures and modify subsequent flowering, with potential consequences for fruit production. In addition, the adverse effects of high temperatures on yield may be amplified by the regulatory influence of fruit load on flowering: in ‘Moncada’ mandarin, fruit presence repressed flowering-related *CiFT*, *CiAP1* and *CiLFY* expression (Munoz-Fambuena et al., 2011), while studies on fruit–meristem dominance linked fruit presence to increased stem auxin transport and inhibited bud release, suggesting the involvement of auxin-mediated signalling in the regulation of bud activity (Marzal et al., 2025). Therefore, a weaker inductive signal resulting from rising temperatures reduces fruit numbers and promotes vegetative growth. This increase in vegetative shoots with the potential to induce flowering (Mesejo et al., 2022) promotes alternate bearing, disrupts the optimal fruit load, and alters the demand for carbon and nutrients throughout the tree.

Overall, available evidence indicates that elevated CO_2_ can modify photosynthetic N allocation in citrus, whereas high temperature and its combination with other environmental stresses can impair photosynthetic functioning, mineral acquisition and reproductive development through responses that depend on thermal intensity and seasonal timing. However, the combined effects of elevated CO_2_ and warming on root nutrient uptake, whole-plant mineral allocation, and nutrient transfer to developing fruit remain largely unresolved. The evidence reviewed here supports effects of elevated CO_2_ and temperature on citrus leaf physiology and growth, but its implications for productive orchards depend on plant developmental stage and experimental conditions. Responses measured in young plants or under controlled conditions should therefore be distinguished from long-term responses in fruit-bearing trees. In particular, changes in photosynthetic performance do not alone establish corresponding changes in nutrient delivery to fruit or fruit quality.

### 3.2 Cold and freezing stress

Alongside the effects of rising mean temperatures, low-temperature episodes remain relevant hazards for citrus production. Seasonal cooling contributes to flowering induction and fruit colour development, but damaging cold and freezing events can impair plant functioning, depending on genotype, acclimation status, and exposure conditions (Vincent et al., 2020; Primo-Capella et al., 2021). Non-freezing cold can restrict carbon assimilation through stomatal and biochemical limitations and alter photosynthetic electron transport. In seven-month-old ‘Valencia’ orange plants grafted onto Rangpur lime, a substrate temperature of 10 °C imposed greater limitations on photosynthesis than atmospheric chilling under the tested conditions, highlighting the importance of the root-zone thermal environment (Santos et al., 2011). More recent work with ‘Valencia Delta Seedless’ grafted onto Carrizo citrange or Citrus macrophylla showed that six weeks at 1 °C modified gas exchange, plant water relations, and aquaporin expression, with rootstock-dependent responses (Primo-Capella et al., 2026). These controlled-environment studies demonstrate physiological responses to chilling, but do not directly establish their consequences for productivity in mature orchards.

Low soil temperatures also influence citrus nutrition by conditioning nutrient acquisition and internal transport. Experimental and modelling work identified soil temperature, root density, nutrient concentration, and availability duration as determinants of N uptake efficiency in citrus (Scholberg et al., 2002). Soil cooling can also reduce the transport of newly absorbed N towards developing fruit, linking the root-zone thermal environment with seasonal N allocation and maturation (Mesejo et al., 2022). These changes should be distinguished from nutritional deficiency, because declining soil-to-fruit N flux also participates in the normal regulation of colour break. At the metabolic level, exposure of five-month-old sour orange plants to −8 °C for 5 h increased nitrate reductase expression and activity, suppressed nitrite reductase expression, and modified the regulation of S-nitrosoglutathione reductase (Ziogas et al., 2013). These findings connect low-temperature responses with nitrate-related metabolism and nitrosative signalling, without demonstrating changes in whole-plant N acquisition or assimilation.

Prolonged fruit development and on-tree retention are particularly relevant in late-maturing citrus cultivars, in which the period from flowering to harvest can exceed 12 months, extending beyond the following spring bloom (Matias et al., 2023). This prolonged presence on the tree spans contrasting seasonal conditions, including winter periods when damaging cold and freezing events may occur. Field evidence shows that freezing can disrupt carbohydrate and respiratory metabolism in citrus fruit. In frost-exposed ‘Valencia Late’ oranges, increased activities of enzymes involved in alternative glycolytic reactions and fermentation were accompanied by a 40% reduction in whole-fruit respiration and accumulation of ethanol and acetaldehyde, indicating a metabolic shift towards fermentation (Falcone Ferreyra et al., 2006). These changes occurred alongside oxidative damage to lipids and proteins and increased membrane leakage, linking freezing injury with alterations in carbon metabolism and cellular integrity. This evidence extends the physiological effects of low temperature to fruit tissues and highlights the relevance of cultivar phenology and harvest timing when assessing seasonal stress exposure.

### 3.3 Water availability and soil conditions

Water scarcity represents a major environmental constraint for citrus production in Mediterranean and other semi-arid regions (Navarro et al., 2023). As soil water availability declines, citrus trees close their stomata to conserve water and restrict transpiration. Although this protective response limits water loss, it also reduces CO_2_ diffusion into the leaves and constrains carbon assimilation (Chen et al., 2023). Simultaneously, soil drying restricts nutrient transport towards the root surface through mass flow and diffusion. Together with reduced root growth and metabolic activity, these changes can limit nutrient acquisition and alter plant mineral status (Sánchez-Bermúdez et al., 2022).

The consequences of water deficit depend strongly on its intensity, duration, and timing within the citrus phenological cycle. In ‘Navelina’ orange trees, flowering and fruit growth were the stages most sensitive to irrigation deficits. A water supply corresponding to approximately 50% of crop evapotranspiration resulted in yield losses of up to 20% when imposed during flowering, compared with approximately 10% during fruit growth and 6% during ripening (García-Tejero et al., 2012). These results demonstrate that similar levels of water deficit can have markedly different consequences depending on the developmental stage affected.

Water-related constraints also concern irrigation-water quality. In dry and hot citrus-growing regions, poor-quality irrigation water can contribute to root-zone salinisation (Ziogas et al., 2021). Salt accumulation is also influenced by soil physical conditions, because impaired infiltration and insufficient leaching can favour salt retention in the root zone. Organic amendments may improve aggregate stability, porosity, and water infiltration, thereby facilitate salt leaching when combined with appropriate water and drainage management (Diacono and Montemurro, 2015). Salinity combines osmotic limitations to water acquisition with the accumulation of potentially toxic Na^+^ and Cl^-^ and disturbances in mineral nutrition. These ions can interfere with the acquisition and balance of nutrients such as K, Ca, Mg, and nitrate, while rootstock-dependent differences in ion exclusion and root-to-shoot transport influence their accumulation in leaves and the resulting injury (Syvertsen and Garcia-Sanchez, 2014). Salinity can also interact with water deficit: in young greenhouse-grown ‘Lima’ sweet orange plants grafted onto ‘Swingle’ citrumelo, combined exposure caused greater reductions in gas exchange and photosynthetic pigment concentrations than either stress alone (Sá et al., 2026). Thus, water availability and salinity should be considered jointly when interpreting citrus physiological and nutritional responses, although the magnitude of combined effects observed in young plants cannot be extrapolated directly to mature orchards.

Climate change is also increasing hydrological variability and the risk of intense precipitation events in Mediterranean environments. Consequently, citrus orchards may experience alternating periods of soil drying and excess water. Although drought and flooding represent opposite soil water conditions, some of their physiological consequences can converge (Chen et al., 2023). During waterlogging, restricted oxygen diffusion into the soil induces root hypoxia or anoxia, limiting aerobic respiration and the energy available for water and nutrient uptake. The resulting reduction in root hydraulic conductance can induce stomatal closure and restrict carbon assimilation even when soil water is abundant. Thus, both drought and flooding can ultimately impair leaf gas exchange and root functioning, although the underlying signals and acclimation mechanisms differ.

Experimental evidence in citrus confirms that waterlogging has direct consequences for mineral nutrition. Flooding reduced nitrogen uptake and modified its distribution among roots, stems, and leaves in citrus seedlings, demonstrating that root hypoxia disrupts both nutrient acquisition and internal nutrient allocation (Martínez-Alcántara et al., 2012). Similarly, 21 days of waterlogging impaired the root mechanisms involved in Fe acquisition in sour orange seedlings. Under these conditions, ^57^Fe uptake declined by approximately 30% in Fe-supplied plants and by 66% in Fe-deficient plants, while its distribution to the aerial organs decreased by approximately 31% and 72%, respectively. These nutritional alterations were accompanied by increased leaf chlorosis and reduced photosynthetic activity (Martínez-Cuenca et al., 2015). These seedling experiments provide direct evidence that waterlogging can impair nutrient acquisition and allocation in citrus. They do not, however, quantify the magnitude or persistence of these effects in mature fruit-bearing trees exposed to episodic flooding under orchard conditions.

Excess water can also modify nutrient availability before acquisition by the roots. Intense rainfall can increase soil erosion in citrus orchards and facilitate the movement of mobile nutrients, particularly nitrate, beyond the root zone (Duan et al., 2020). Prolonged waterlogging, in turn, decreases soil oxygen availability and redox potential, altering microbial processes and nutrient transformations and ultimately restricting nutrient acquisition by roots (Zhang et al., 2025). Waterlogging can alter tissue mineral composition and cause nutrient deficiencies or imbalances. In two citrus rootstocks, flooding generally decreased Ca, K, Mg, Na, and Cl concentrations in leaves and roots, although the magnitude of the response depended on the genotype and tissue examined (García‐Sánchez et al., 2007). This pattern is consistent with evidence from other crops indicating that oxygen deprivation restricts ATP production and nutrient uptake, and that nutrient deficiency, rather than the accumulation of toxic mineral concentrations, may be a major cause of growth inhibition under waterlogging (Steffens et al., 2005).

Waterlogging may also impose a combined abiotic and biotic constraint on citrus roots. In addition to the direct effects of oxygen deprivation on root functioning, poorly drained and saturated soils can favour *Phytophthora* root rot (Graham and Feichtenberger, 2015). In container-grown grapefruit trees, *P. nicotianae* infection combined with six weeks of flooding reduced root hydraulic conductance more than infection under non-flooded conditions, demonstrating that these constraints can jointly impair root function (Chaudhary et al., 2016). In Sicily, *P. inundata* was identified alongside *P. nicotianae* in declining mature citrus trees, and combined inoculation caused more severe root rot than *P. nicotianae* alone in citrus saplings (Bua et al., 2025). These findings suggest that prolonged soil saturation may expose citrus roots to both physiological impairment and pathogen-related damage, with disease risk depending on drainage, pathogen presence, and rootstock susceptibility.

Moreover, physiological recovery after drainage may be incomplete and strongly dependent on the citrus rootstock. Differences among citrus genotypes have been observed in their capacity to regenerate the root system and recover photosynthesis, stomatal conductance, and transpiration after waterlogging (Partiya et al., 2018; Pérez-Jiménez and Pérez-Tornero, 2021).

## 4 Physiological and nutritional regulation of citrus fruit quality

### 4.1 Carbon–nitrogen balance and source–sink relationships

Citrus fruit development depends on the coordinated acquisition and allocation of carbon and mineral nutrients. In this context, carbon refers primarily to photosynthetically assimilated carbon incorporated into carbohydrates, which provide both energy and the structural carbon skeletons required for fruit growth and metabolism (Sadka et al., 2019). Mature leaves constitute the main source organs, exporting much of the newly assimilated carbon through the phloem in the form of sucrose, the major translocated sugar in citrus trees. Developing shoots, roots, flowers, and fruits can act as competing sinks, whose relative strength changes throughout the annual cycle (Goldschmidt, 1999). Following its arrival in the fruit, sucrose is unloaded and metabolised, thereby providing energy and carbon skeletons that support fruit growth and the synthesis of structural and metabolic compounds (Khan et al., 2023). Beyond this metabolic role, sugars also participate in developmental signalling and therefore do not merely determine carbohydrate accumulation. In citrus fruit, changes in sucrose availability and metabolism have been associated with the regulation of ripening, colour development, and carotenoid biosynthesis (Iglesias et al., 2001; Zhang et al., 2012).

Maintaining a coordinated balance between carbon supply and N availability is essential in citrus because it influences photosynthetic activity, vegetative growth, and resource allocation to reproductive organs (Huang et al., 2022; Mesejo et al., 2022). Rather than representing a fixed whole-tree C:N ratio, this balance emerges from the dynamic coordination of carbon assimilation with N uptake, remobilisation, and allocation throughout the annual cycle. Within this system, N plays a dual role: it supports the photosynthetic apparatus and therefore source capacity, while also contributing to the growth and metabolism of vegetative and reproductive sinks (Liao et al., 2019; Zhao et al., 2025b).

This coordination between carbon allocation and mineral nutrition also involves other nutrients, because fruit sink strength depends on nutrient availability, whereas carbohydrate supply supports nutrient acquisition and utilisation within developing tissues. In sweet orange, K supply increased both source and sink strength, enhanced symplastic sucrose loading, and promoted carbon flow from source leaves to developing fruits, providing a direct mechanistic link between mineral nutrition and carbohydrate partitioning (Wu et al., 2024). This coordination involves multiple nutrients acting at different points of the source–sink pathway, from photosynthetic carbon assimilation to phloem transport and fruit growth.

Mineral nutrients beyond N can influence source functioning and resource allocation, while their availability is conditioned by the soil environment. In calcareous soils, reactions with Ca can reduce P solubility, while alkaline pH limits Zn and Mn availability (Obreza et al., 2012). Interactions among nutrients can also affect plant physiological functioning. In young navel orange plants, high P supply under Mg deficiency accelerated chlorophyll degradation, photosynthetic impairment, and oxidative damage in older leaves while promoting new shoot growth (Hu et al., 2026). Although no climatic stress was imposed, this response demonstrates that nutrient imbalance can affect photosynthetic source capacity and the distribution of nutrients among organs. Under high irradiation and temperature, additional Mg supply enhanced antioxidant regulation and reduced oxidative damage in young lemon trees (Boaretto et al., 2020). Boron supply can also modify the balance between carbon assimilation and water conservation: in ‘Carrizo’ citrange seedlings, irrigation with 5 mg L^-1^ B, compared with 0.11 mg L^-1^ increased foliar ABA accumulation and reduced stomatal conductance, transpiration, and photosynthesis, while improving leaf water status during subsequent drought and recovery after rewatering (Tasa et al., 2025). These nutritional interactions are particularly relevant under climatic stress, because soil drying can restrict nutrient movement towards roots and alter nutrient availability (Franco‐Navarro et al., 2025), while excessive heat can impair root growth and nutrient-acquisition processes (Mishra et al., 2019). These findings link nutritional status with source functioning and stress responses, although they do not establish corresponding effects on carbon allocation to fruit in mature orchards.

Against this background, climate change may disturb C–N coordination by affecting carbon and N dynamics through different and potentially asynchronous mechanisms. As discussed above, elevated CO_2_ can initially enhance carbon assimilation and vegetative biomass production, increasing the demand of developing shoots for both carbohydrates and N, although this response may weaken as photosynthetic acclimation develops (Keutgen and Chen, 2001). Concurrent elevated CO_2_ may partly offset warming-related limitations to carbon assimilation, as observed in young ‘Ambersweet’ orange trees (Vu et al., 2002). Consequently, the proposed mismatch depends on the balance between carbon supply and changing sink demand, rather than on an inevitable decline in photosynthesis; its expression will depend on photosynthetic acclimation, thermal exposure, and water and nutrient availability. At the same time, high air temperatures can constrain photosynthesis and reproductive development, whereas warmer soils may sustain root activity and N uptake for longer periods during the annual cycle (Otero et al., 2008; Roccuzzo et al., 2017). Warmer soil conditions may also prolong N transport to developing fruits later in the season, as demonstrated by Mesejo et al. (2022). These seasonal effects of warmer soils should be distinguished from the impairment of root functioning under excessive temperatures, as discussed above; temperature responses depend on genotype and exposure conditions.

The relevance of this temporal coordination becomes particularly evident during citrus fruit maturation. Using ^15^N labelling and controlled soil cooling, Mesejo et al. (2022) demonstrated that declining soil temperature reduced the translocation of newly absorbed N from roots to fruits. This reduction in soil-to-fruit N flux coincided with the onset of fruit N remobilisation and external colour change. Conversely, maintaining warmer soil conditions prolonged N transport to the fruit and delayed colour break. These findings demonstrate that soil temperature can regulate citrus ripening indirectly by modifying seasonal N fluxes, thereby establishing a mechanistic connection between the root environment, mineral nutrition, and fruit phenology. In a warm climate, prolonged root-to-fruit N transport later in the season could therefore alter the temporal coordination between carbon accumulation and N remobilisation, with potential consequences for the progression of fruit maturation.

Alongside these changes in nutrient supply and transport, environmental stress can also modify carbohydrate allocation through the regulation of fruit sink metabolism. In citrus, drought-induced sugar accumulation has been associated with increased expression of genes involved in sucrose metabolism and sugar transport in the segment membranes and juice sacs (Khan et al., 2023). More recently, Khan et al. (2025) identified the CsMYB1–CwINV6 regulatory module in ‘Nanfeng’ tangerine (*Citrus reticulata*). Drought and ABA treatments increased the expression of both genes, and CsMYB1 was shown to bind directly to the CwINV6 promoter and activate its expression. Transient overexpression or silencing of either gene in citrus fruits increased or decreased glucose and fructose accumulation, respectively. These findings establish a mechanistic connection between stress-responsive transcriptional regulation, cell-wall invertase expression, and fruit sugar accumulation, indicating that carbohydrate responses to stress depend on changes in sink metabolism as well as source carbon supply. Such evidence provides a basis for investigating climate-responsive regulation of carbon allocation in citrus. In this context, the concept of climate-responsive optimisation of carbon partitioning to sinks (CROCS), developed by Lou et al. (2025) and discussed by Zhao et al. (2025a), offers a potential framework for exploring whether stress-responsive regulation of sucrose metabolism could help sustain reproductive sink strength under warming. Although the citrus evidence described above concerns drought rather than heat, it identifies regulatory mechanisms relevant to this research direction. The applicability of CROCS to citrus requires experimental validation, particularly regarding the coordination of fruit carbohydrate metabolism with carbon supply and N allocation.

Changes in N availability can influence the timing and intensity of vegetative growth in citrus. In potted ‘Newhall’ sweet orange seedlings, reducing N supply delayed summer-flush emergence and shortened the flush period (Li et al., 2026a). Nitrogen-responsive cytokinin signalling provides a potential mechanism linking nutrient availability to shoot activity, based on evidence from Arabidopsis and broader plant research (Landrein et al., 2018; Sakakibara, 2021). This mechanism has not been demonstrated in citrus in the context considered here. Competition for assimilated carbon between vegetative and reproductive organs has been demonstrated in ‘Moncada’ mandarin, where fruit development limited shoot growth through photoassimilate limitation (Martínez-Alcántara et al., 2015). Together, these findings suggest that prolonged N availability could modify the timing and intensity of vegetative sink demand. If this coincides with reduced carbon assimilation, competition for available photoassimilates may intensify; however, whether these conditions shift allocation away from developing fruits requires experimental validation. Climate-induced C imbalance should thus be considered as a potential temporal and spatial mismatch among carbon assimilation, N acquisition and optimisation, and the changing demands of vegetative and reproductive sinks. **Figure 3** illustrates this proposed disruption of C–N coordination.

**Figure 3:**
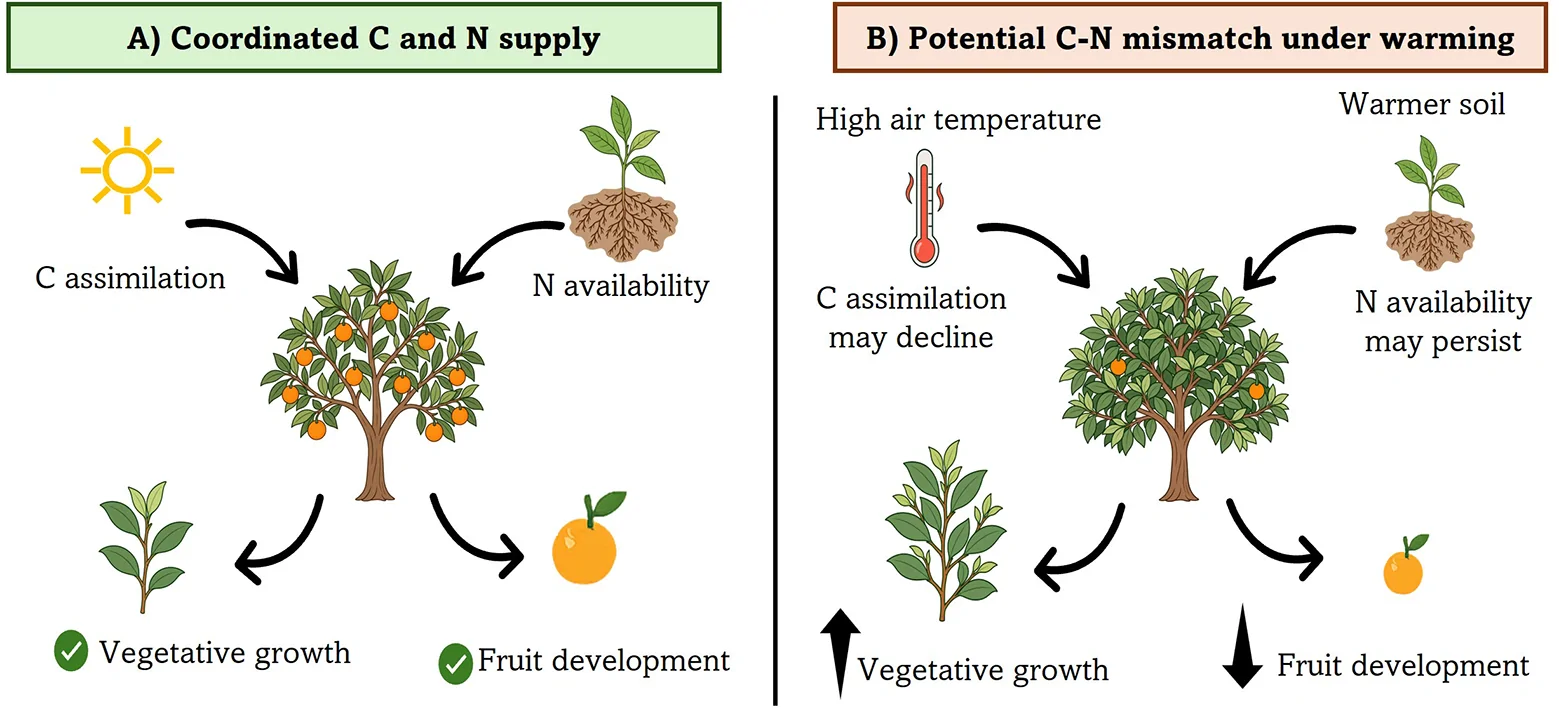
Conceptual representation of carbon–nitrogen coordination and its potential disruption in citrus under warming. **(A)** Coordinated carbon assimilation and nitrogen availability supporting vegetative growth and fruit development. **(B)** Potential carbon–nitrogen mismatch under warming, whereby carbon assimilation may decline while nitrogen availability persists, potentially favouring vegetative growth over fruit development. This original illustration synthesises citrus evidence on CO2-responsive carbon assimilation and growth, seasonal N dynamics, and competition for photoassimilates between vegetative and reproductive organs (Keutgen and Chen, 2001; Roccuzzo et al., 2017; Martínez-Alcántara et al., 2015; Mesejo et al., 2022; Li et al., 2026a). Together, these studies support the physiological basis of the proposed disruption in the timing and distribution of carbon and N among competing sinks. Increased vegetative growth and reduced fruit development represent potential integrated outcomes requiring validation under concurrent warming and elevated CO_2_, rather than an inevitable decline in whole-tree carbon assimilation.

### 4.2 Mineral nutrition, fruit development, and physiological disorders

The consequences of source–sink regulation for fruit development depend not only on carbon allocation, but also on how climate-induced changes in root activity and nutrient availability affect mineral uptake and delivery to the fruit. Developing citrus fruits act as major nutrient sinks, although the timing and magnitude of mineral accumulation vary among elements and developmental stages. In four sweet orange cultivars, the total amounts of N, P, K, Ca, and Mg accumulated in the fruit increased as fruit biomass expanded, while their concentrations generally declined because dry matter accumulated faster than minerals (Paramasivam et al., 2000). Thus, a decline in mineral concentration during fruit growth may reflect dilution rather than reduced nutrient uptake or deficiency. Environmental fluctuations can further alter the balance between fruit expansion and nutrient supply. Drought and high temperatures may reduce root activity, soil water availability, and transpirational flow, restricting nutrient acquisition and delivery to developing organs (Hussain et al., 2023). When water uptake resumes after a dry period, rapid pulp expansion may exert pressure on a rind that cannot adjust at the same rate, increasing susceptibility to splitting (Krajewski et al., 2022). Such mismatches between water uptake, fruit growth, and nutrient delivery may contribute to physiological disorders even without visible symptoms of whole-tree nutrient deficiency.

Fruit splitting is a multifactorial disorder that cannot be attributed solely to environmental conditions or to a single nutrient deficiency. Its occurrence also depends on cultivar susceptibility, peel anatomy and mechanical properties, fruit load, water availability, mineral nutrition, and the relative growth rates of the pulp and rind (Kaur et al., 2025). In the splitting-prone citrus hybrid ‘Ehime Kashi 34’, K fertilisation reduced fruit splitting and modified peel anatomical, mineral, and metabolic traits (Jiao et al., 2022). Although this study did not directly address climate change, its findings indicate that mineral nutrition can modulate fruit resistance to splitting and may therefore influence citrus vulnerability under increasingly variable water regimes. Similarly, foliar application of chelated Ca to ‘Okitsu No. 58’ increased Ca accumulation in the peel, modified pectin metabolism and antioxidant activity, and reduced fruit cracking. However, the response varied with the applied concentration (Wang et al., 2024). Under field conditions, the effectiveness of nutritional interventions also depends on environmental factors controlling the absorption and subsequent translocation of foliar-applied nutrients (Fernández and Brown, 2013). Climate change may therefore not only increase fruit susceptibility to splitting but also modify the effectiveness and predictability of the nutritional strategies used to mitigate it.

Other physiological disorders are more directly associated with the aerial environment and may therefore become increasingly relevant under climate change. Sunburn represents a particularly clear example, as climate warming is expected to intensify the thermal stress responsible for its development in citrus fruit (Park et al., 2023). Excessive solar radiation combined with elevated fruit-surface temperature can cause peel discoloration and different degrees of cellular damage. In field-grown ‘Rio Red’ grapefruit, reflective treatments slightly reduced fruit temperature and sunburn incidence, whereas an antitranspirant increased fruit temperature and resulted in a higher proportion of sunburned fruit (Rodriguez et al., 2019). Together, these findings identify fruit-surface temperature as a direct driver of sunburn and support the use of reflective treatments as a potential adaptation strategy under increasingly warm conditions.

Although direct evidence linking climate change to an increased incidence of citrus physiological disorders remains limited, more frequent heat events and fluctuations in water availability may intensify the mechanisms underlying fruit splitting and sunburn. Their occurrence will ultimately depend on the interaction among environmental conditions, mineral nutrition, fruit developmental stage, and cultivar susceptibility.

### 4.3 Ripening and compositional quality

Beyond effects on fruit growth and physiological disorders, climate change can also influence reproductive performance, fruit maturation, and the compositional traits that determine citrus fruit quality. Experimental evidence from greenhouse-grown ‘Shiranuhi’ mandarin illustrates the breadth of these effects. High-temperature treatments imposed from 20 to 270 days after full bloom affected fruit retention, primary metabolism, and rind coloration. Under the most intense daytime-warming treatment, cumulative exposure above 30 °C increased from 123 to 1,543 h, while fruit set declined from 23.3 to 12.4% and yield decreased from 25.9 to 7.7 kg tree^-1^, despite enhanced growth of the remaining fruit. Total free-sugar concentration also declined from 114.0 to 101.8 mg mL^-1^, possibly reflecting greater carbohydrate utilisation under sustained heat. Moreover, combined daytime and night-time warming reduced citric acid concentration from 12.4 to 7.9 mg mL^-1^, a response consistent with increased metabolic consumption of organic acids, while delaying rind coloration and altering secondary-metabolite accumulation (Kim et al., 2021). These responses demonstrate the effects of imposed warming on fruit-bearing trees under greenhouse conditions, but their magnitude should not be extrapolated directly to open-field orchards with different cultivars and environmental conditions.

Water availability also influences fruit growth and composition and should be considered alongside temperature when interpreting climate-related quality changes. In ‘Lane Late’ sweet orange, deficit irrigation during stage III slightly reduced fruit weight and juice percentage while increasing total soluble solids and titratable acidity, without significantly altering the maturity index (Pérez-Pérez et al., 2009). These responses are consistent with restricted fruit hydration and expansion, but changes in soluble solids and acidity alone do not distinguish altered sugar and acid metabolism from concentration effects associated with reduced fruit water content. Increases in soluble solids under water deficit should therefore not necessarily be interpreted as enhanced carbohydrate accumulation, as they may partly result from reduced water content and the consequent concentration of juice solutes.

Long-term field evidence further illustrates the context-dependent effects of temperature on citrus fruit composition. Across six growing seasons, days above 35 °C during early summer were associated with lower juice and sugar contents and higher organic acid concentrations under arid conditions. By contrast, under Mediterranean conditions, temperatures between 27 and 33 °C during late summer and early autumn were associated with lower citric acid concentrations (Mesejo et al., 2024). Whereas the greenhouse experiment in ‘Shiranuhi’ demonstrates responses to imposed warming, the multi-season field study identifies associations between thermal conditions and fruit composition. Together, these studies support the importance of thermal intensity and developmental timing, although the magnitude of the reported responses cannot be generalised across citrus production systems.

Elevated atmospheric CO_2_ may also influence citrus fruit production and composition, although direct evidence remains limited. In a long-term experiment with well-watered and adequately fertilised sour orange trees, exposure to approximately 700 ppm CO_2_ increased fruit number by around 74%, individual fruit fresh weight by 4%, and juice vitamin C concentration by 5% during the final years of the study (Idso et al., 2002). Although these findings provide long-term evidence from adult, fruit-bearing trees, their extrapolation to commercial citrus orchards is constrained by the species studied and the adequate water and nutrient supply maintained during the experiment. Responses in commercial scion cultivars, particularly under combined climatic stresses, therefore require further evaluation. Moreover, because comparable evidence for sugars, organic acids, pigments, and fruit mineral composition remains limited, the long-term consequences of rising CO_2_ for citrus fruit composition and nutrient density remain poorly understood.

Overall, the evidence indicates that citrus fruit-quality responses to climatic conditions depend on exposure intensity, duration, developmental timing, and growing conditions. Temperature, water availability, and atmospheric CO_2_ should therefore be considered jointly, although their combined effects on fruit composition require further experimental evaluation. Resolving these interactions will help improve predictions of fruit-quality changes and guide adaptation strategies.

### 4.4 Redox regulation and antioxidant-related fruit quality

Environmental effects on citrus metabolism also depend on the capacity to coordinate antioxidant defenses under stress. In plants exposed to combined drought and heat, the greater tolerance of Carrizo citrange relative to Cleopatra mandarin was associated with coordinated activities of superoxide dismutase (SOD), ascorbate peroxidase (APX), catalase (CAT), and glutathione reductase (GR), together with maintenance of a favourable reduced-to-oxidised glutathione ratio (GSH/GSSG) (Zandalinas et al., 2017). By contrast, Cleopatra showed increased SOD activity but inefficient GR activation and a lack of CAT and APX activities under the stress combination, a response associated with greater oxidative stress and susceptibility. This contrast highlights that effective antioxidant protection depends on coordinated ROS detoxification and maintenance of redox balance, rather than on increased activity of an individual enzyme. Such genotype-dependent responses help explain differences in tolerance to combined climatic stresses, although their consequences for fruit antioxidant composition require direct evaluation.

Evidence from citrus fruit shows how environmental stress can disrupt metabolism and quality, while also revealing that biochemical responses do not necessarily confer protection. In frost-exposed ‘Valencia late’ oranges, oxidative damage and increased membrane leakage accompanied a shift toward fermentative metabolism: whole-fruit respiration declined by 40%, ethanol and acetaldehyde accumulated, and titratable acidity increased by 39%, reducing the maturity index by 24% (Falcone Ferreyra et al., 2006). The influence of environmental exposure can also extend beyond harvest. In ‘Tango’ and ‘Nadorcott’ mandarins and ‘Valencia’ oranges, greater preharvest exposure to temperatures below 5 °C or relative humidity below 30% was associated with altered peel fatty acid metabolism and greater chilling injury during storage at 2 °C. Notably, the fatty acid unsaturation ratio correlated positively with injury severity, cautioning against interpreting increased unsaturation as universally protective (Lozano-Omeñaca et al., 2026). A similar distinction between compositional change and functional protection applies to antioxidants. Although temperature-dependent carotenoid accumulation has been demonstrated in cultured citrus juice sacs (Yungyuen et al., 2018), its protective significance requires separate assessment. In mandarin peel, higher β-cryptoxanthin and violaxanthin concentrations were associated with greater singlet oxygen-quenching capacity and lower postharvest chilling injury, whereas vitamin C concentration was positively associated with injury susceptibility (Rey et al., 2020). Thus, antioxidant-related quality traits should be interpreted alongside their functional activity and the metabolic condition of the tissue. These complementary findings support a connection between environmental exposure, metabolic integrity, and fruit susceptibility, although evidence from field comparisons, isolated tissues, and postharvest storage has different implications for fruit responses under orchard conditions.

## 5 Strategies to strengthen soil–plant functioning and nutritional resilience

Enhancing the resilience of established citrus orchards requires understanding how management can sustain soil functionality, root activity, nutrient acquisition, and fruit development within the existing scion–rootstock combination. This section considers three complementary perspectives: soil management and amendments that support soil–root functioning; biostimulants and exogenous signalling compounds that may modify plant, root, and rhizosphere responses to environmental stress; and the potential integration of these approaches. Treatments involving biostimulants and signalling compounds are interpreted according to the evidence for stress mitigation, pretreatment-induced protection, or persistent priming. The potential benefits and interactions of these strategies are assessed alongside their dependence on genotype and orchard conditions, implementation costs, and practical feasibility.

### 5.1 Soil management and amendments supporting soil–root functioning

Climate-resilient citrus production depends in part on maintaining soil conditions that support root activity and mineral nutrient acquisition under environmental variability. Water deficit can restrict root activity and nutrient acquisition, whereas waterlogging limits soil aeration and root respiration (Syvertsen and Garcia-Sanchez, 2014). When contrasting stresses occur in succession, their effects on root functioning cannot necessarily be inferred from responses to each stress individually (Sánchez-Bermúdez et al., 2022). Soil water availability, aeration, and nutrient supply must therefore be considered together when assessing root-zone functioning and citrus productivity (Ziogas et al., 2021). Within this context, soil-focused practices such as organic amendments, mulching, and cover crops may help regulate water availability, nutrient retention and cycling, soil structure, and biological activity. The main practices evaluated in citrus and the functions through which they may contribute to soil–plant resilience are summarised in **Table 2**.

**Table 2:** Selected soil-management practices supporting soil–root functioning in citrus under variable climatic conditions.

Strategy	Study context: location; duration; stress	Main evidence and limitations	Main function	Reference
Mulching				
Hardwood-oak mulch	Florida, USA; 3 years; HLB-affected trees, no climatic stress imposed.	Soil moisture +25–88%; SOM + 49%. Responses in healthy trees and climatic-stress tolerance not established.	Water conservation and soil fertility	Hallman et al. (2023)
Rice-straw mulch	Two citrus orchards; semi-arid Mediterranean climate; almost 3 years. no climatic stress imposed.	Soil temperature −0.5–1.2 °C; SOC mass fraction increased 10% more than under bare soil; plant heat tolerance not directly assessed.	Soil thermal regulation and C accumulation	Visconti et al. (2024)
Organic mulch	Eastern Sicily, Italy; 3 seasons; full irrigation versus 50% ETc during fruit-development stage II.	Soil water content up to +27% under deficit irrigation; benefit specific to the evaluated irrigation conditions.	Water conservation	Vanella et al. (2025)
Compost amendment				
Rice straw-sewage sludge compost	Valencia, Spain; adult ‘Tango’ orchard on clay loam; 2 growing seasons; flood irrigation, no climatic stress imposed.	Increased SOM, organic N, and available P and K; greater plant uptake not directly established by these soil measurements.	Soil fertility	Rodríguez-Carretero et al. (2026)
Compost amendment	Hau Giang, Vietnam; 3 years; no climatic stress imposed.	Mean yield: 20.8 versus 15.3 t ha^-1^ with compost + NPK versus NPK alone; climatic-stress tolerance not tested.	Soil fertility and productivity	Dang et al. (2022)
Citrus-branch compost	Guangxi, China; subtropical monsoon climate; acidic Acrisol; 2 years (2022–2024); no climatic stress imposed.	Enhanced N mineralization and P-cycling potential; yield +34.6%; climatic-stress tolerance requires separate assessment.	Nutrient cycling and productivity	Li et al. (2026a)
Cover crops and integrated organic management				
Cover crops	Southwest Florida, USA; tropical savanna climate; ≈30 months; no climatic stress imposed.	Changes in soil C and N cycling in the upper 10 cm; deeper root-zone responses not established.	Nutrient cycling	Castellano-Hinojosa et al. (2023)
Integrated organic management	Eastern Sicily, Italy; 15 commercial orchards; summer 2021 and winter 2021–2022, no climatic stress imposed.	Greater microbial diversity and nutrient-cycling potential; cycling rates not directly measured.	Microbial functional potential	Taguali et al. (2025)
Integrated organic management: cover crops, residue retention, and no tillage	Mediterranean citrus orchard; 21 years; management comparison.	SOC stocks +16.5 t C ha^-1^; contribution of individual practices not isolated.	Soil C accumulation	Novara et al. (2019)

SOC, soil organic carbon; SOM, soil organic matter; HLB, huanglongbing. Percentage changes are relative to the corresponding study controls. Benefits to soil properties or productivity should be distinguished from demonstrated tolerance to climatic stress.

Increasing soil organic matter is, therefore, a central component of this strategy because it promotes aggregate formation and improves pore structure. These changes favour water infiltration and storage, create a more suitable environment for root and microbial activity, and support nutrient retention and cycling (Murphy, 2015). Organic-matter restoration may also increase plant-available water because its effect on water retention is generally greater at field capacity than at the permanent wilting point, although the magnitude of this response depends on soil texture, initial organic-matter content, and local environmental conditions (Lal, 2020).

The physical benefits of organic-matter management are complemented by the direct protection provided by mulching. By insulating the soil surface, mulches create a more stable root-zone microenvironment, whereas the gradual decomposition of organic materials extends these benefits by contributing to longer-term improvements in soil functioning (Saputra et al., 2025). Over nearly three years, rice-straw mulching in Mediterranean citrus orchards reduced mean topsoil temperature by 0.5–1.2 °C and increased soil organic carbon (SOC) accumulation by 10% relative to bare soil (Visconti et al., 2024). In a three-year field trial in Florida, hardwood-oak mulch increased soil moisture by 25–88%, depending on season, and soil organic matter by 49% compared with unmulched controls (Hallman et al., 2023). These responses should be interpreted in the context of the sandy soil studied (Oldsmar fine sand) and HLB-affected trees, rather than extrapolated directly to orchards with different soil textures, climatic conditions, or tree health status. Similarly, in a three-year experiment with orange trees, regulated deficit irrigation reduced water inputs by 24%, while organic mulching increased soil water content by up to 27% relative to bare soil under the same irrigation regime (Vanella et al., 2025). The improvement in soil moisture associated with mulching should therefore be distinguished from the water savings achieved through irrigation management. Together, these studies illustrate changes in soil temperature and moisture under the conditions evaluated; their agronomic value also depends on the resulting plant water status, yield, and fruit quality.

Organic inputs can also improve nutrient availability and support crop productivity, although responses depend on soil conditions and application duration. In a commercial Mediterranean citrus orchard, successive applications of rice-straw–sewage-sludge compost increased soil organic matter and organic N, enhanced P and K availability, while maintaining tree nutritional status and fruit production despite a reduction in mineral P inputs (Rodríguez-Carretero et al., 2026). Similarly, in a three-year pomelo field trial in Vietnam, compost applied at 5 t ha^-1^ year^-1^ alongside a common NPK fertilisation programme produced a mean yield of 20.8 t ha^-1^, compared with 15.3 t ha^-1^ under NPK alone. Biochar and lime were evaluated as separate treatments. These results support soil-fertility and productivity benefits, although tolerance to climatic stress was not directly tested (Dang et al., 2022). The contribution of organic inputs to nutrient availability depends not only on their nutrient content, but also on the biological processes governing nutrient release and cycling.

These nutritional benefits are closely linked to soil biological activity. Soil microorganisms produce extracellular enzymes that depolymerise organic compounds, while mineralisation and solubilisation release nutrients from organic and sparingly available pools, facilitating their subsequent acquisition by plant roots (Jacoby et al., 2017; Singh et al., 2022). Consistent with this role, compost applications temporarily stimulated soil biological indicators in the Mediterranean citrus orchard studied by Rodríguez-Carretero et al. (2026). Evidence from commercial Mediterranean citrus orchards also showed that soil total carbon was among the environmental variables shaping microbial community composition, while organic management was associated with greater microbial diversity, more resilient interaction networks, and a stronger representation of functions related to nutrient cycling (Taguali et al., 2025). More direct evidence was obtained in a two-year citrus-orchard experiment, where compost produced from pruning residues enhanced the microbial functional potential associated with N mineralisation and P cycling, increased soil nutrient availability, and raised fruit yield by 34.6% relative to conventional chemical fertilisation (Li et al., 2026b).

The relationship between root nutrition and microbial activity is bidirectional, because nutrient release from roots can also shape microbial colonisation. Evidence from Arabidopsis, discussed by Lu et al. (2026), shows that endodermal barrier integrity regulates glutamine leakage, thereby influencing bacterial chemotaxis and spatial colonisation of root surfaces. Together, these findings support a role for soil microorganisms in linking organic inputs with nutrient cycling and root-zone functioning. However, changes in microbial diversity or functional potential should be distinguished from measured nutrient fluxes and plant uptake, and do not establish that microbial changes alone explain the observed productivity benefits.

The balance between organic inputs and microbial decomposition also determines whether improvements in soil functioning are accompanied by sustained carbon storage, providing a potential contribution to climate-change mitigation. Globally, approximately 60 Gt C year^-1^ (nearly half of the carbon annually assimilated by terrestrial vegetation) is transferred to soils through litter and root-derived inputs (Lal, 2008; Pausch and Kuzyakov, 2018). However, carbon input should not be equated with long-term sequestration, as a substantial proportion is rapidly processed by microorganisms and returned to the atmosphere through respiration. Orchard management can shift this balance by increasing organic inputs through cover crops, residue retention and organic amendments. In a commercial Florida citrus orchard, 30 months of cover cropping altered soil C and N concentrations, mineralisation, greenhouse-gas emissions, and N-cycling microbial communities, although these effects were largely confined to the upper 0–10 cm of soil (Castellano-Hinojosa et al., 2023). Evidence of longer-term accumulation was reported in a Mediterranean orange orchard, where 21 years of organic management based on cover crops, residue retention, and no tillage increased soil organic carbon stocks by 16.5 t C ha^-1^, corresponding to an average sequestration rate of 0.78 t C ha^-1^ year^-1^ (Novara et al., 2019). Because this management system combined cover crops, residue retention, and no tillage, the increase in soil carbon cannot be attributed to any one practice alone. This long-term accumulation should also be distinguished from the shorter-term changes in surface-soil carbon and nutrient cycling observed in other experiments.

### 5.2 Biostimulants and signalling compounds for stress mitigation and physiological priming

Under field conditions, plants frequently encounter overlapping or successive environmental constraints, whose combined effects cannot be inferred from their responses to each stress individually (Sánchez-Bermúdez et al., 2022). These responses are further shaped by the order in which stresses occur and by the developmental and genetic background of the plant (Jiang et al., 2025). Under combined drought, heat, and high irradiance, Carrizo citrange maintained lower leaf temperatures and showed less disruption of the photosynthetic apparatus than Cleopatra mandarin. Carrizo also exhibited stronger activation of genes involved in photosystem II repair and better preservation of the corresponding proteins, consistent with improved photosynthetic recovery after stress (Balfagón et al., 2022). These findings highlight leaf cooling and photosystem repair as mechanisms associated with genotype-dependent tolerance to combined stresses.

Beyond responses to concurrent stresses, previous environmental exposure can also modify how plants respond to a later stress event through acclimation or cross-protection. Stress priming describes the preparation induced by an initial stimulus that alters responsiveness to subsequent stress, whereas persistent stress memory involves the retention of this altered responsiveness beyond the initial stimulus (Hilker et al., 2016; Crisp et al., 2016). Evaluating treatment-induced priming therefore requires distinguishing applications during stress, pretreatments followed by stress, and responses retained after treatment withdrawal. Pretreatment alone does not establish persistent memory because residual treatment activity or sustained changes in nutrition and growth may also improve subsequent tolerance.

Evidence from environmental stress sequences illustrates the potential for acquired tolerance in citrus. Repeated dehydration–rehydration cycles increased the resistance of ‘Ponkan’ mandarin seedlings to later water limitation (Toro-Herrera et al., 2023). Drought-induced changes in ‘Valencia’ sweet orange were retained in vegetative buds and influenced the response of the resulting grafted plants to subsequent water deficit (de Oliveira Sousa et al., 2022). In ‘Flame’ grapefruit, moderate drought before freezing enhanced cold tolerance through antioxidant activity, osmolyte accumulation, and carbohydrate adjustment (Iqbal et al., 2025). These findings support a role for previous environmental experience in citrus stress responses, but do not establish that biostimulants applications produce comparable persistent effects in mature orchards.

The capacity of citrus plants to retain effects of previous environmental exposure raises the possibility that physiological preparedness could also be deliberately induced through agricultural management, using exogenous compounds or biostimulants that activate stress-responsive pathways before subsequent stress exposure (Kerchev et al., 2020). Roots may represent a relevant target for these interventions because they integrate environmental cues through interconnected hormonal, redox, and Ca^2+^-signalling networks that coordinate local and systemic stress responses in fruit crops (Xu and Wang, 2026). Mineral nutrition may also influence physiological preparedness through its roles in stress-responsive signalling, ion homeostasis, osmotic adjustment, membrane stability, and antioxidant defense (Gupta et al., 2024), although these functions alone do not demonstrate nutrient-induced priming. The following examples are grouped into non-microbial biostimulants and related treatments, microbial biostimulants, and exogenous signalling compounds, with their interpretation guided by treatment timing and the evidence for persistence after withdrawal (**Table 3**).

**Table 3:** Experimental designs and evidence for stress mitigation, pretreatment-induced protection, and persistent stress memory in citrus.

Strategy	Study context	Main evidence and limitations	Main function	Reference
Non-microbial				
Seaweed extract	‘Hamlin’ nursery trees; deficit irrigation; applications during stress.	Improved water-use efficiency and maintained growth; persistence after withdrawal not tested.	Water-use efficiency and growth maintenance	Spann and Little (2011)
Seaweed and fishmeal extracts	Mature ‘Valencia’ orchard; 2 seasons; irrigation regimes including water deficit.	Improved growth, yield, and fruit-quality attributes; persistent priming not tested.	Agronomic performance	Ennab and Alam-Eldein (2020)
Potassium fulvic acid	Trifoliate orange seedlings; pretreatment followed by salinity.	Reduced injury and modified rhizosphere enzymes and bacterial populations; residual treatment effects not excluded.	Stress protection and rhizosphere modulation	Zhang et al. (2023)
Microbial				
Bacterial inoculation	Cleopatra mandarin; inoculation 10 days before combined heat and high light.	Prevented leaf abscission and improved photosynthesis; continued colonization may contribute to protection.	Photosynthetic protection	Terán et al. (2025)
Mycorrhizal association	Roots connected to adult grapefruit trees; established symbiosis before 15 weeks of localised soil drying.	Reduced fine-root mortality; remaining roots received water, so whole-tree drought tolerance was not established.	Local root survival	Espeleta et al. (1999)
*Bacillus subtilis*	Outdoor container-grown ‘Fortune’ trees; 3 seasonal applications; no defined abiotic stress.	Improved growth, photosynthesis, fertiliser-^15^N use efficiency, and P and K contents; stress priming not evaluated.	Physiological and nutritional support	Pérez-Piqueres et al. (2025)
Exogenous signalling compounds				
Salicylic acid	Young ‘Lima’ orange plants; drought, salinity, or both; applications before and during stress.	Improved physiological and biochemical responses; pretreatment and concurrent effects not isolated.	Stress mitigation	Sá et al. (2026)
Melatonin	Mexican and Persian lime; drought; timing relative to stress onset unclear.	Reduced oxidative damage and electrolyte leakage; separate pretreatment and persistent memory not established.	Antioxidant and membrane protection	Jafari et al. (2022)
H_2_O_2_ and NO donor	Sour orange; root pretreatment followed by salinity.	Systemic antioxidant and proteomic responses with reduced oxidative damage; persistent memory not independently established.	Systemic physiological preparation	Tanou et al. (2009, Tanou et al., 2012)
Proline	UF 914 citrus hybrid; 1 month of foliar applications before freezing at −6 °C.	Reduced oxidative damage and preserved photochemical function; persistence independently of residual effects not tested.	Photosynthetic and antioxidant protection	Aucique-Perez et al. (2026)
NO and H_2_S donors	Root pretreatment for 2 days; 5-day acclimation interval; 21 days of PEG-induced water deficit.	Responses consistent with retained preparedness; residual donor activity not excluded.	Redox-mediated physiological preparation	Ziogas et al. (2015)

Non-microbial biostimulants include seaweed extracts, humic substances, protein hydrolysates, and amino-acid-based formulations that can influence physiological processes associated with plant stress tolerance (Ma et al., 2022). In citrus, available examples differ in the timing of treatment relative to stress exposure. An *Ascophyllum nodosum* extract improved water-use efficiency and maintained growth in ‘Hamlin’ sweet orange nursery trees under deficit irrigation (Spann and Little, 2011). Evidence also extends to mature orchards: in a two-season field experiment with 15-year-old ‘Valencia’ orange trees, seasonal foliar applications of seaweed and fishmeal extracts improved growth, yield, and several fruit-quality attributes across irrigation regimes that included deficit irrigation (Ennab and Alam-Eldein, 2020). These studies support benefits during water limitation, but neither tested whether enhanced stress responsiveness persisted after treatment withdrawal. By contrast, potassium fulvic acid applied to trifoliate orange seedlings before salt exposure reduced subsequent injury, increased rhizosphere enzyme activities, and favoured beneficial Bacillus populations (Zhang et al., 2023). This pretreatment design is consistent with a preparatory effect involving soil–plant–microbiome interactions but does not isolate persistent stress memory from residual product activity or sustained changes in the rhizosphere. Thus, improved performance during treatment and protection following pretreatment should be distinguished from evidence of enhanced responsiveness after withdrawal of the initial stimulus. Silicon supplementation has also been associated with improved ion homeostasis, membrane stability, and antioxidant protection in citrus (de Souza Junior et al., 2025). However, benefits observed while silicon remains available support stress mitigation without establishing persistent priming after treatment withdrawal.

Microbial biostimulants provide examples of both pretreatment before a defined stress challenge and repeated applications throughout the growing season. In Cleopatra mandarin, inoculation with *Pseudomonas putida* KT2440 or *Novosphingobium* sp. HR1a 10 days before combined high-temperature and high-light exposure prevented leaf abscission and improved photosynthetic performance (Terán et al., 2025). Bacterial colonisation was still detected after exposure, indicating that protection following pretreatment could involve continued microbial activity. Evidence from mature field-grown trees also supports a protective role of mycorrhizal association under localised soil drying. Using root compartments connected to adult grapefruit trees, Espeleta et al. (1999) established mycorrhizal and non-mycorrhizal roots before withholding irrigation from the upper compartments for 15 weeks. Mycorrhizal roots exhibited lower fine-root mortality, although the remaining root system continued to receive water; these results therefore concern local root survival rather than whole-tree drought tolerance. In a complementary experiment, Pérez-Piqueres et al. (2025) applied *Bacillus subtilis* formulations three times during the growing season to three-year-old ‘Fortune’ mandarin trees grown in outdoor containers. Individual strains improved growth and photosynthetic performance and increased fertiliser-^15^N use efficiency and plant P and K contents. This study supports physiological and nutritional benefits during a seasonal inoculation programme, rather than demonstrating priming before a defined stress challenge. Neither experiment tested enhanced stress responsiveness after withdrawal of the microbial stimulus; continued microbial activity could therefore contribute to the observed benefits.

Exogenous signalling compounds provide experimental approaches to distinguish stress mitigation from preparation for subsequent stress. Salicylic-acid applications initiated before stress and continued during exposure improved physiological and biochemical responses of young ‘Lima’ sweet orange plants grafted onto ‘Swingle’ citrumelo under water deficit, salinity, and their combination (Sá et al., 2026). Because applications overlapped with stress exposure, this design supports stress mitigation but does not isolate preparedness retained after treatment withdrawal. Melatonin also reduced drought-induced oxidative damage and electrolyte leakage and modulated antioxidant regulation in Mexican and Persian lime plants (Jafari et al., 2022). However, the reported methods do not clearly specify a separate pretreatment period before drought, so these findings should not be interpreted as demonstrating persistent priming. More explicit pretreatment designs include the application of H_2_O_2_ or the nitric oxide donor sodium nitroprusside to sour orange roots before NaCl exposure, which stimulated systemic antioxidant and proteomic responses in leaves and reduced oxidative damage (Tanou et al., 2009, Tanou et al., 2012). Similarly, a month-long foliar proline application programme preceding exposure to −6 °C reduced oxidative damage and helped preserve photosynthetic photochemical function in the citrus hybrid UF 914 (Aucique-Pérez et al., 2025). These studies support protection against subsequent stress, but do not establish its persistence independently of residual treatment effects. A clearer temporal separation was reported by Ziogas et al. (2015), who pretreated citrus roots with sodium nitroprusside or sodium hydrosulfide, donors of NO and H_2_S, respectively, for two days, followed by a five-day acclimation period before 21 days of PEG-induced water deficit. This design provides evidence consistent with retained physiological preparedness and supports the proposed role of redox signalling in citrus priming (Molassiotis et al., 2016). Nevertheless, an interval between treatment and stress does not by itself exclude residual treatment effects; demonstrating persistent stress memory requires controls that distinguish retained changes in plant responsiveness from ongoing compound activity.

Despite these promising findings, evidence for biostimulant-induced priming in citrus remains limited. Future studies should distinguish transient treatment effects from persistent priming by incorporating treatment withdrawal, recovery periods, and subsequent stress challenges. Testing these responses under recurrent or sequential stresses in mature field-grown trees, while integrating physiological, nutritional, molecular, and rhizosphere measurements, will be essential to determine their persistence, agronomic relevance, and potential application in climate-resilient citrus production.

### 5.3 Integration of practices, trade-offs, and practical feasibility

Soil-focused practices and biostimulant interventions may provide complementary contributions to citrus resilience through their effects on the root-zone environment, nutrient acquisition, and plant physiology. Field evidence shows that combining soil moisture management with alginate oligosaccharide applications can improve soil properties, root growth, photosynthetic performance, and fruit production, although outcomes differ among treatment combinations (Li et al., 2024). Benefits should nevertheless not be assumed across management contexts. In citrus seedlings supplied with high phosphorus, mycorrhizal inoculation produced genotype- and CO_2_-dependent growth responses, including growth depression in sour orange under ambient CO_2_ (Jifon et al., 2002). Integration therefore requires considering the soil and nutritional environment as well as treatment timing; applications initiated before stress but continued during exposure do not isolate pretreatment effects (Sá et al., 2026). These findings provide a rationale for evaluating combined practices, rather than assuming their superiority. Demonstrating synergy requires comparisons with the individual interventions and explicit assessment of their interaction, while possible adverse responses should also be evaluated.

The suitability of these approaches depends on soil texture, initial organic-matter content, climate, and orchard developmental stage. Benefits associated with organic-matter restoration and water retention are context-dependent (Lal, 2020), while treatment responses in young plants cannot be directly extrapolated to mature productive trees. Practical trade-offs also differ among strategies. Organic amendments require suitable materials, transport, and distribution, whereas cover crops require establishment and ongoing management. Biostimulants incur product and application costs, particularly when repeated treatments are needed. Pretreatment-based approaches additionally depend on whether application can be timed before the relevant stress event. These considerations favour locally adapted management rather than uniform combinations across citrus-growing regions.

Economic assessment should therefore complement agronomic evaluation. Existing citrus studies indicate that adaptation costs can be partly offset by operational savings and that adaptation can be compatible with maintaining profit efficiency. In a partial-budget analysis of Florida orange groves, estimated savings from eliminating mowing offset approximately 49% of the additional annual cost of cover-crop adoption (Chakravarty and Wade, 2023). An analysis of citrus farms in South Africa did not detect an overall reduction in profit efficiency associated with the climate-adaptation strategies evaluated (Joseph et al., 2021). Although these findings do not establish the economic returns of biostimulant-based adaptation, they highlight the need to assess both expenditure and potential benefits. For the practices considered here, these benefits include marketable yield, fruit quality, avoided stress-related losses, and possible input savings. Multi-season field assessments are needed to determine their net economic value under variable stress exposure and input and fruit prices.

## 6 Research gaps and future directions

Three research priorities emerge from the evidence reviewed. First, multi-year studies in mature citrus orchards are needed to determine how combined and sequential climatic stresses affect the coordination between carbon assimilation, nutrient acquisition and remobilisation, and vegetative and reproductive demand. Measurements should span successive phenological stages and jointly assess physiological responses, nutrient allocation, fruit development, and physiological disorders. A central unresolved question is whether a temporal mismatch between carbon supply and N availability contributes to fruit-quality responses, and how this relationship varies among scion–rootstock combinations, soil properties, and stress sequences.

Second, the effectiveness of soil-focused and plant-focused interventions should be evaluated individually and in combination under defined climatic constraints. Field comparisons should establish whether combined practices provide additional benefits, have overlapping effects, or produce antagonistic responses. Their assessment should include fruit yield and quality, input requirements, costs, and feasibility across contrasting orchard conditions, rather than relying solely on short-term physiological improvements.

Third, persistent treatment-induced priming requires specific experimental validation in citrus, particularly in mature trees. Studies should distinguish applications during stress from pretreatments followed by a subsequent challenge and include treatment withdrawal, an appropriate interval, and controls for residual compound activity or continued microbial association. These designs are needed to determine whether altered stress responsiveness persists beyond the initial treatment and whether it provides benefits across subsequent stress events or growing seasons.

Addressing these priorities would provide a stronger basis for linking climate-driven physiological changes with nutrient dynamics and fruit outcomes in citrus. This knowledge is needed to guide adaptation strategies suited to specific orchard conditions and to assess their capacity to sustain productivity and fruit quality across successive seasons.
